# Development of a one-step multiplex RT-qPCR method for rapid detection of bovine diarrhea viruses

**DOI:** 10.3389/fcimb.2024.1540710

**Published:** 2025-01-28

**Authors:** Dequan Yang, Li Ma, Zhongping Yang, Xianchao Yang, Jian Wang, Houbin Ju, Chunguang Lu, Yonggang Weng, Heping Zhao, Haixiao Shen, Xin Li, Feifei Ge, Xiaoxu Wang, Xiujuan Wu, Meng Xiang, Guidan Feng, Congsheng Tang, Shixin Huang, Hongjin Zhao

**Affiliations:** ^1^ Veterinary Diagnostic Center, Shanghai Animal Disease Control Center, Shanghai, China; ^2^ Department of Technological Research and Development, Hunan Guanmu Biotech Co., Ltd, Changsha, China; ^3^ Department of Veterinary Laboratory, Jinshan District Animal Disease Control Center, Shanghai, China

**Keywords:** calf diarrhea, multiplex real-time quantitative PCR (mRT-qPCR), clinical detection, method, epidemiological surveillance

## Abstract

**Introduction:**

Viral calf diarrhea poses a significant challenge to the cattle industry worldwide due to its high morbidity and mortality rates, leading to substantial economic losses. The clinical symptoms associated with various diarrhea pathogens often overlap, complicating accurate diagnosis; thus, there is an urgent need for rapid and precise diagnostic methods to improve prevention and treatment efforts. In this study, we developed a one-step multiplex reverse-transcription quantitative real-time polymerase chain reaction (mRT-qPCR) that enables the simultaneous detection of three key viral pathogens responsible for calf diarrhea: bovine kobuvirus (BKoV), bovine astrovirus (BoAstV), and bovine torovirus (BToV). However, development of accurate and rapid methods to distinguish these three viruses is helpful for the early detection, disease surveillance, and control of viral calf diarrhea.

**Methods:**

Specific primers and minor groove binder (MGB)-based probes were designed targeting the 3D region of BKoV, ORF1 region of BoAstV, and N region of BToV. The sensitivity, specificity, and reproducibility ability were evaluated for the mRT-qPCR. Further, 80 bovine fecal samples were subjected to the mRT-qPCR, and the results were verified using conventional reverse-transcription PCR (RT-PCR) or PCR methods and sequencing methods.

**Results:**

This novel method demonstrated high sensitivity and specificity,achieving a detection limit of 24 copies/mL for each pathogen. Furthermore, the assay exhibited excellent reproducibility, with coefficients of variation below 1.5%, a strong linear correlation (R^2^ > 0.996), and an amplification efficiency between 90% and 110%. Validation with 80 clinical samples from both diarrheic and non-diarrheic cattle across four farms in Shanghai showed a high degree of concordance with RT-PCR, with positive detection rates for BKoV, BoAstV, and BToV at 28.75%, 8.75%, and 3.75%, respectively, highlighting the predominance of BKoV and BoAstV. Notably, this study represents the first identification of BKoV, BoAstV, and BToV in the Shanghai region.

**Discussion:**

The mRT-qPCR is a robust, rapid, and simple tool for identifying viral pathogens associated with calf diarrhea, facilitating the development of effective prevention and control measures that are vital for the future sustainability of the cattle industry.

## 1 Introduction

Calf diarrhea is a prevalent gastrointestinal disease in young cattle and a major contributor to economic losses for cattle producers worldwide (Kim et al., 2021; Jessop et al., 2024; Lorenz and Trefz, 2024). The condition is often attributed to single or co-infections by various enteric pathogens, including viruses, bacteria, and protozoa (Çelik and Kozat, 2024; Jessop et al., 2024). In recent years, viral pathogens have garnered increasing attention for their role in the etiology of calf diarrhea (Papageorgiou et al., 2024). Several viruses, such as bovine rotavirus (BRV), bovine coronavirus (BCoV), bovine viral diarrhea virus (BVDV), and bovine adenovirus (BAdV), among others, have been recognized as key causative agents (Gomez and Weese, 2017). However, limited research has been dedicated to the detection methods for bovine kobuvirus (BKoV), bovine astrovirus (BoAstV), and bovine torovirus (BToV), despite their emerging importance in calf diarrhea outbreaks.

Bovine kobuvirus (BKoV) was first identified in 2003 in Japan in HeLa cell cultures containing calf serum, and it has since been detected across 13 countries spanning four continents, indicating its global prevalence (Hao et al., 2021; Li et al., 2024). Studies suggest that BKoV is a potential causative agent of calf diarrhea, particularly in neonatal cattle (Chang et al., 2014; Işidan et al., 2019; Wang et al., 2020; Hao et al., 2021; Li et al., 2024). Bovine astrovirus (BoAstV), first identified in diarrheic calf stool samples in the United Kingdom in 1978, has similarly been detected in multiple countries (Zhu et al., 2022). Recent research has revealed BoAstV not only in the gastrointestinal system but also in brain tissues of animals with neurological symptoms, indicating its potential to cause both enteric and neurotropic diseases (Mansour et al., 2021; Roach and Langlois, 2021). Co-infections involving BoAstV, particularly with BRV and BKoV, are common, with the highest co-infection rate reported at 66.67% (Zhu et al., 2021). Bovine torovirus (BToV), first identified during a U.S. diarrhea outbreak in 1979, is also known to cause diarrhea in calves and adult cattle, leading to significant economic losses due to weight loss and reduced milk production (Zhao et al., 2021; Castells et al., 2024).

The clinical symptoms of calf diarrhea caused by these viral pathogens are often indistinguishable, and co-infections further complicate diagnosis. Traditional diagnostic methods, such as pathogen isolation, are insufficient for rapid and accurate identification. Differential diagnosis typically requires specific antigen detection assays. While singleplex real-time PCR assays have been developed for the rapid detection of some diarrhea-related pathogens (Lüthi et al., 2018; Zhao et al., 2021; Liu et al., 2022), these methods are resource-intensive and inefficient for processing multiple pathogens simultaneously. Thus, there is an urgent need for a rapid, efficient, and comprehensive diagnostic method capable of detecting multiple viral pathogens in a single assay (Song et al., 2023; Meng et al., 2024).

In this study, we focused on three major diarrhea-causing viruses—BKoV, BoAstV, and BToV—that present with similar clinical symptoms. We developed a one-step multiplex reverse-transcription quantitative real-time PCR (mRT-qPCR) assay targeting highly conserved genomic regions of these viruses. The goal was to create a diagnostic tool that is faster and more accurate than conventional molecular assays. The new mRT-qPCR assay was validated through assessments of specificity, sensitivity, and reproducibility, and it demonstrated robust performance, offering a reliable solution for the simultaneous detection of BKoV, BoAstV, and BToV in clinical samples.

## 2 Materials and methods

### 2.1 Primers and probes

Specific primers and probes targeting the genomes of bovine kobuvirus (BKoV), bovine astrovirus (BoAstV), and bovine torovirus (BToV) were meticulously designed using Primer Express 3.0.1 software (Applied Biosystems, Foster City, United States). These designs were based on conserved regions identified within all available sequences for the aforementioned viruses sourced from GenBank. The selected genomic regions for amplification included the 3D, open reading frame 1 (ORF1), and nucleocapsid (N) gene segments specific to BKoV, BoAstV, and BToV, respectively. The synthesis of these primers and probes was performed by Shanghai Saiheng Biotech Co., Ltd. Additionally, all primers utilized in conventional reverse transcription polymerase chain reaction (RT-PCR) or polymerase chain reaction (PCR) assays in this study were referenced from previously published reports (Jeoung et al., 2011; Mohamed et al., 2017; Wang et al., 2019). The detailed sequences of primers and probes can be found in **Table 1**.

**Table 1:** Sequences of primers and probes designed for the mRT-qPCR assay targeting BoAstV, BKoV, and BToV, including their fluorescent labeling for multiplex detection.

Pathogens	Name	Gene	Length(bp)	Sequence (5′-3′)	Position	Genbank number
BKoV	F	3D	62	TCCCGCCAACAAAGGTTCT	7811-7872	OK019998
	R			TGAGGAAGGTGACGTCGTAGAG		
	P			6-FAM-ACTTYCCTGACTCYTCCA-MGB		
BoAstV	F	ORF1	63	CGCACAGGCACTTGCTACTC	613-675	KM035759
	R			ATGCCACTCCCAATAYAAACAAGAT		
	P			HEX-ATGATGAGGCATCCC-MGB		
BToV	F	N	60	CAGCCACAGCCACAAGTAGTG	27661-27720	MN882587
	R			CCGTGGTTGAAAGCCCATA		
	P			Cy5-CTATGCCCATTCARTATC-MGB		

“F” is forward primer, “R” is reverse primer, “P” is probe.

### 2.2 Virus strains and clinical samples

To ensure diverse viral detection, positive samples were screened for BKoV (Jeoung et al., 2011), BoAstV (Mohamed et al., 2017) and BToV (Mohamed et al., 2017), and other related viruses, including bovine viral diarrhea virus (BVDV) (Mohamed et al., 2017), bovine rotavirus (BRV) (Mohamed et al., 2017), bovine parvovirus (BPV) (Wang et al., 2019), bovine norovirus (BNoV) (Mohamed et al., 2017), bovine coronavirus (BCoV) (Mohamed et al., 2017). These screenings employed singleplex conventional RT-PCR or PCR techniques, previously established in our laboratory, and the identity of the viruses was further confirmed through DNA sequencing conducted by Shanghai Saiheng Biotech Co., Ltd.

A total of 80 bovine fecal samples were collected from four dairy farms located in Shanghai in 2024. The sample collection was stratified based on clinical presentation and age: 40 samples were sourced from calves exhibiting diarrhea, while another 40 samples were obtained from healthy, non-diarrheic calves. In terms of age distribution, 40 fecal samples were collected from calves younger than 3 months, and 40 samples were retrieved from older calves aged between 7 to 14 months. The calves presenting diarrhea had shown these symptoms persistently for over three days, with fecal samples being collected during the critical period of days 3 to 5 post-onset of diarrhea. Specific details pertaining to all collected samples are outlined in **Table 2**. Upon collection, all fecal specimens were promptly stored at -80°C to preserve nucleic acid integrity until further processing.

**Table 2:** Detailed information on the bovine fecal samples collected, including farm name, sample numbers, cattle age in months, and associated clinical symptoms.

Farm name	Number of samples	Months of cattle	Clinical symptoms
A	10	< 3	Diarrhea
	10	< 3	Non-diarrhea
B	10	7~14	Diarrhea
	10	7~14	Non-diarrhea
C	10	7~14	Diarrhea
	10	7~14	Non-diarrhea
D	10	< 3	Diarrhea
	10	< 3	Non-diarrhea
Total	80		

### 2.3 RNA extraction

For RNA extraction, fecal samples were first homogenized with phosphate-buffered saline (PBS) using a vortex mixer for a duration of a few minutes to ensure uniform suspension. Following homogenization, the mixtures were subjected to centrifugation at 12,000 × g for 10 minutes at 4°C to pellet debris. Nucleic acids were subsequently extracted from the supernatant using the Magnetic Viral DNA/RNA kit (Guanmu Biotechnology, China, Cat#GM10001-16), employing the Automated Nucleic Acid Extractor GM (Guanmu Biotechnology, China) in accordance with the manufacturer’s protocol. The concentration and quality of the extracted viral nucleic acids were assessed using a NanoDrop 2000c spectrophotometer (Thermo Scientific, USA), and all samples were stored at -80°C until further analysis via multiplex real-time quantitative PCR (mRT-qPCR).

### 2.4 Construction of standard plasmid

To facilitate accurate quantification in our multiplex reverse-transcription quantitative real-time polymerase chain reaction (mRT-qPCR) assay, we synthesized target fragments corresponding to the mRT-qPCR target sequences for bovine astrovirus (BoAstV), bovine kobuvirus (BKoV), and bovine torovirus (BToV). These fragments were subsequently cloned into a pUC57 vector using the TA cloning method. The successful insertion of the target sequences was verified through DNA sequencing conducted by Sangon Biotech (China). The concentration of the constructed standard plasmid was measured using a NanoDrop-2000c spectrophotometer (Thermo Scientific, USA), and the plasmid copy number was calculated using the following Equation 1:


(1) $$Copy\;number\left(copies/\mu L\right)=\frac{\left(6.02\times 10^{23}\right)\times \left(\frac{ng}{\mu L}\times 10^{-9}\right)}{DNA\;Length\times 660}$$

Here, 6.02×10^23^ is Avogadro’s constant, the DNA length is the total base pair count of the cloned sequence plus the vector, and 660 is the average molecular weight of a base pair in nucleic acids. Subsequent to the quantification and copy number calculation, the standard plasmid was subjected to a 10-fold serial dilution protocol. This dilution ranged from an initial concentration of 2.4×10^9^ copies/μL down to 2.4×10^0^ copies/μL, thereby creating a comprehensive range of standards for calibration within the mRT-qPCR assays. Each dilution was tested in triplicate to ensure the consistency and reliability of the standard curves, which were essential for quantifying viral load in clinical samples. Standard curves were generated from these dilutions, and the corresponding equations were derived to confirm the reliability and accuracy of the dilution series.

### 2.5 Multiplex real-time quantitative PCR

The mRT-qPCR reaction conditions were optimized for the simultaneous detection of BKoV, BoAstV, and BToV. Each 25 µL reaction mixture contained 12.5 µL of 2×GM One-Step Probe RT-qPCR MasterMix (Guanmu Biotechnology, China), 1 µL of 25×GM Enzyme Mix (Guanmu Biotechnology, China), 0.2 µL of dNTPs, 0.2 µL of each primer set (10 µM), and 0.1 µL of each probe (10 µM) corresponding to the target fragment. Additionally, 5 µL of nucleic acid template was added to the mixture, and the final volume was adjusted using enzyme-free water. The mRT-qPCR assay was conducted using the SLAN-96S machine (Hongshi Tech, China) under the following thermal cycling conditions: reverse transcription at 50°C for 15 minutes, followed by an initial denaturation step at 95°C for 2 minutes. This was followed by 40 amplification cycles of 95°C for 15 seconds (denaturation) and 60°C for 30 seconds (annealing). A final step at 25°C for 10 seconds concluded the reaction. During the annealing phase, data acquisition occurred on three fluorescent channels: FAM (blue), HEX (green), and Cy5 (red), corresponding to the probes targeting BKoV, BoAstV, and BToV, respectively.

### 2.6 Sensitivity

The sensitivity of the mRT-qPCR assay was determined by testing 10-fold serial dilutions of a standard plasmid ranging from 2.4×10^9^ to 2.4×10^0^ copies/µL. The limit of detection (LOD) was defined as the lowest concentration at which consistent amplification was observed. Standard curves were generated based on the threshold cycle (Ct) values obtained from the serial dilutions, and the experiment was repeated three times to ensure reproducibility. The amplification efficiency (E) was calculated using the formula: E = 10^(-1/slope) - 1, where the slope was derived from the linear regression of the standard curve. The calculated efficiencies for each target were evaluated to ensure that they fell within the acceptable range of 90-110%, confirming the assay’s high sensitivity.

### 2.7 Specificity

To evaluate the specificity of the mRT-qPCR assay, reactions were performed using viral RNA or DNA templates from both target and non-target viruses. The non-target viruses included bovine viral diarrhea virus (BVDV), bovine rotavirus (BRV), bovine parvovirus (BPV), bovine norovirus (BNoV), and bovine coronavirus (BCoV). Additionally, a negative control consisting of nuclease-free water was included. Each reaction was repeated three times to ensure reliability. The assay demonstrated no cross-reactivity with non-target viruses, confirming its high specificity for BKoV, BoAstV, and BToV, ensuring that only the desired targets were detected.

### 2.8 Repeatability

The repeatability and stability of the mRT-qPCR assay were evaluated by calculating the intra-assay and inter-assay coefficients of variation (CVs). For this purpose, 10-fold serial dilutions of the standard plasmid were tested in at least three replicates in both intra- and inter-assay conditions. The CVs were calculated based on the Ct values obtained for each dilution, ensuring that the variability within and between assays was minimal. Intra-assay CVs were calculated from replicates within a single run, while inter-assay CVs were derived from replicates across multiple runs. Both CVs were consistently below 3%, indicating the assay’s robust repeatability and reliable performance across different experimental conditions.

### 2.9 Clinical application

To evaluate the clinical utility of the developed mRT-qPCR assay, viral RNA was extracted from 80 bovine fecal samples and analyzed using the newly established multiplex assay. These fecal samples were collected from calves presenting both diarrheic and non-diarrheic symptoms. The results of the mRT-qPCR assay were compared with those obtained using previously established singleplex conventional RT-PCR or PCR methods described in earlier studies (Jeoung et al., 2011; Mohamed et al., 2017; Wang et al., 2019).

### 2.10 Statistical analysis

The mRT-qPCR results for each fecal sample were recorded either as positive or negative for each pathogen and categorized according to calf age (< 3 months and 7~14 months) and clinical symptoms (diarrhea or non-diarrhea). The data were analyzed using the SPSS Statistics 25.0 software package for Windows (SPSS, Chicago, IL, USA). The associations between diarrhea and each pathogen were calculated for all ages together and for each age group separately using Pearson’s chi-squared test or Fisher’s exact test, as appropriate. P value of < 0.05 was considered to be statistically significant.

## 3 Results

### 3.1 Preparation of primers, probes and plasmid standards

The development of the mRT-qPCR assay commenced with the design and synthesis of specific primers and probes for the detection of three bovine viruses: bovine kobuvirus (BKoV), bovine astrovirus (BoAstV), and bovine torovirus (BToV). The sequences of these primers and probes are listed in **Table 1**. Target-specific probes were labeled with distinct fluorescent dyes—FAM for BKoV, HEX for BoAstV, and Cy5 for BToV—allowing for the simultaneous multiplex detection of these viral agents. Standard plasmids, representing a range of concentrations from 2.4×10^9^ to 2.4×10^0^ copies/µL, were prepared for use as templates in the mRT-qPCR reactions, enabling quantitative analysis of viral loads in subsequent experiments.

### 3.2 Establishment of the standard curve for the mRT-qPCR

**Figure 1:**
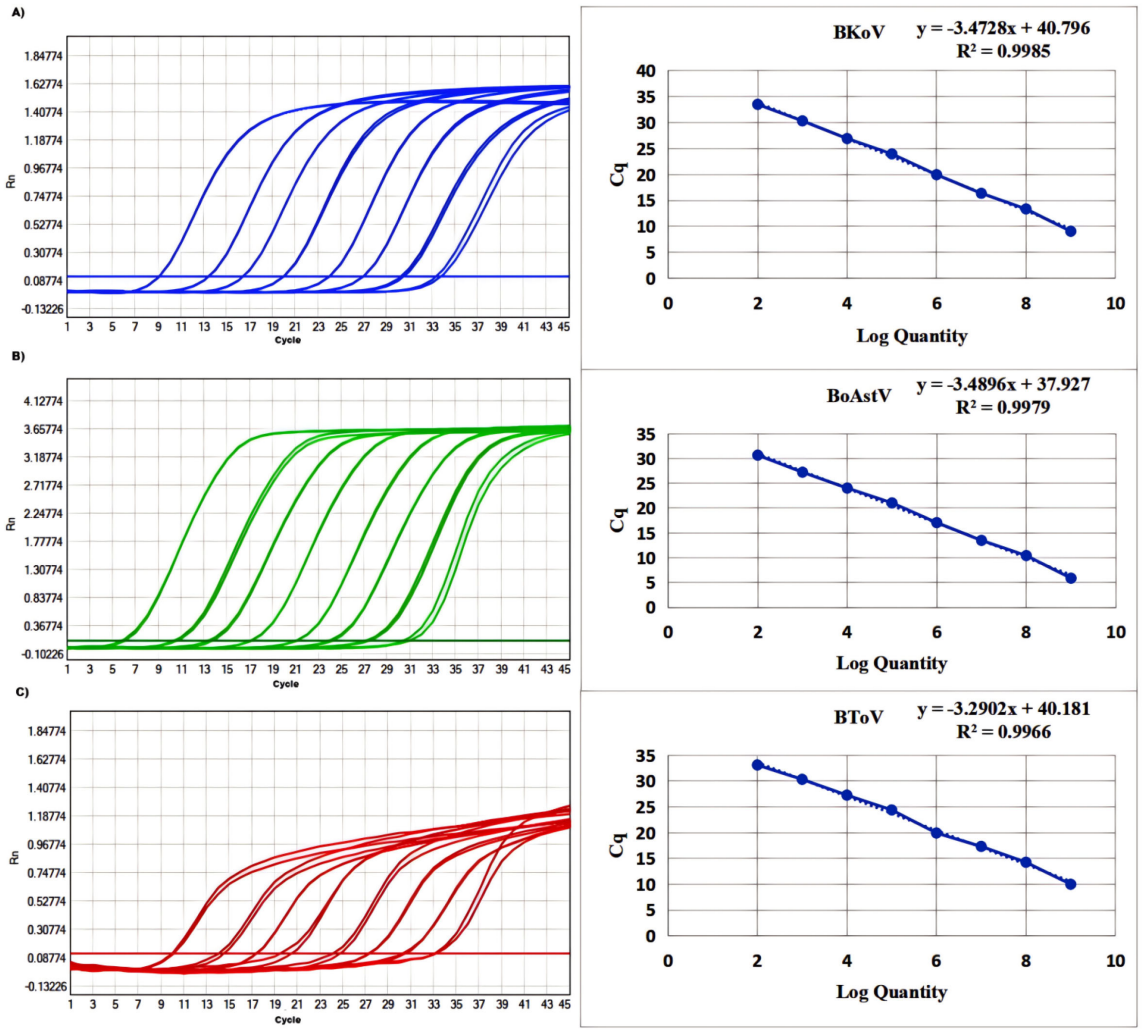
Amplification and standard curves of **(A)** BKoV, **(B)** BoAstV, and **(C)** BToV. The standard curve was evaluated using standards containing 2.4×10^9^ to 2.4×10^2^ copies/μL. BKoV means bovine kobuvirus, BoAstV means bovine astrovirus, and BToV means bovine torovirus.

To assess the quantitative capability of the mRT-qPCR assay, standard curves were generated through a series of 10-fold serial dilutions of the standard plasmid, ranging from 2.4×10^9^ to 2.4×10^2^ copies/µL. The resulting standard curves exhibited excellent amplification efficiency and high correlation coefficients, validating the effectiveness of the assay. The parameters obtained were as follows: for BKoV, the R² value was 0.9985 with an efficiency (E) of 97.92%; for BoAstV, the R² value was 0.9979 with an E value of 97.58%; and for BToV, the R² was 0.9966 with an E of 99.44% (**Figure 1**). These high R² values indicate a strong linear relationship between the log of the input copy number and the corresponding Ct values, confirming that the plasmid standards are capable of providing reliable quantification across the specified concentration range.

### 3.3 Sensitivity of the mRT-qPCR

**Figure 2:**
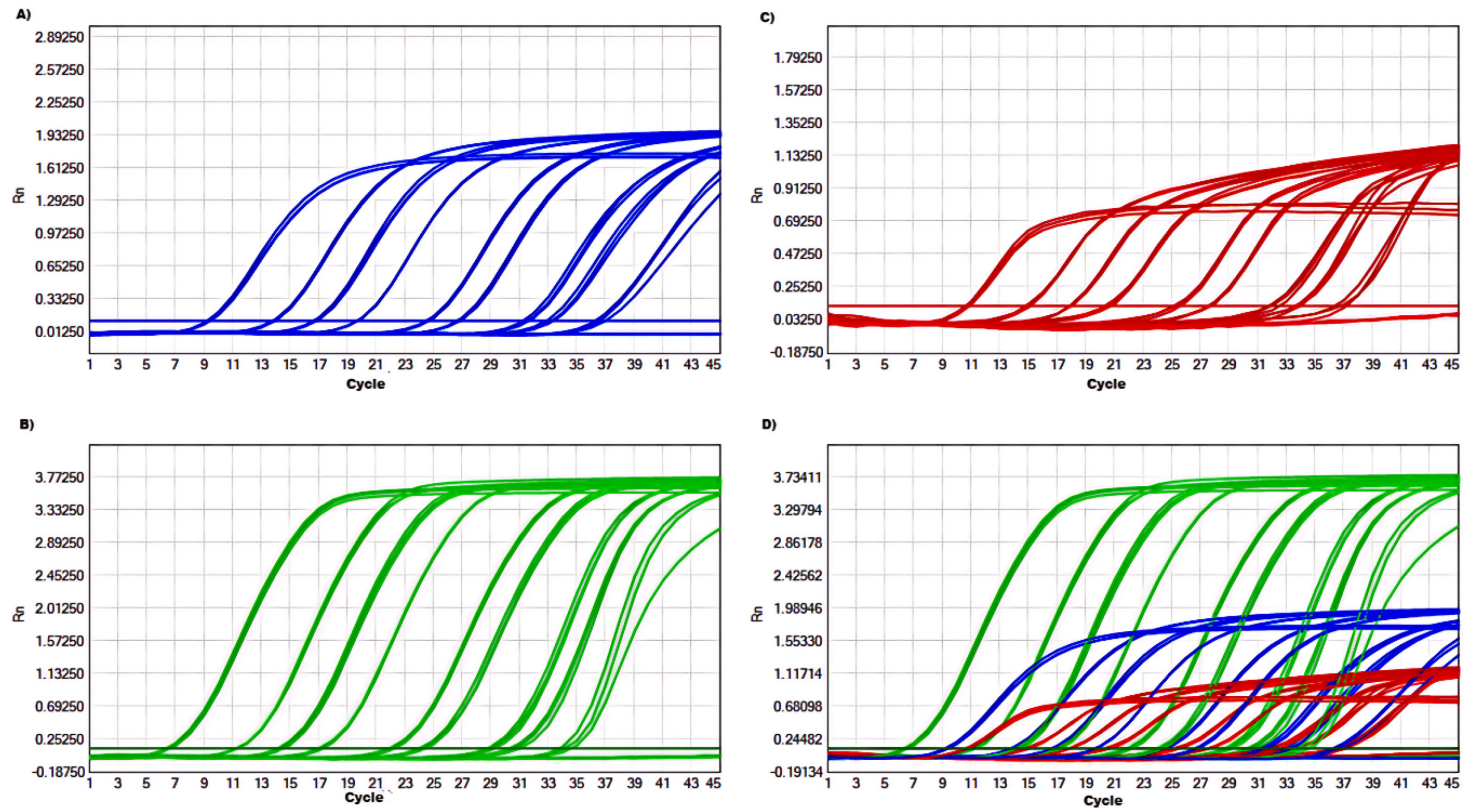
Sensitivity of the mRT-qPCR assay: **(A-D)** amplification curves (X-axis: Cycle, Y-axis: Rn) of BKoV, BoAstV and BToV for each plasmid standard of concentrations with 2.4×10^9^ to 2.4×10^0^ copies/μL. BKoV means bovine kobuvirus, BoAstV means bovine astrovirus, and BToV means bovine torovirus.

To determine the limit of detection (LOD) for the mRT-qPCR assay, a range of viral copy numbers (2.4×10^9^ to 2.4×10^0^ copies/µL) were tested in triplicate. The assay demonstrated a high level of sensitivity, with the ability to detect as few as 24 viral copies for each of the three target viruses (BKoV, BoAstV, and BToV). The corresponding quantification cycle (Cq) values ranged from 34 to 37, indicating reliable detection even at low concentrations (**Figures 2A–D**; **Table 3**). This high sensitivity confirms that the mRT-qPCR assay is capable of detecting low viral loads, making it suitable for early-stage diagnosis and epidemiological studies of viral diarrhea in calves.

**Table 3:** Limit of detection (LOD) assessment for the mRT-qPCR assay, showing results from testing standard plasmid dilutions ranging from 2.4 × 10^¹^ to 2.4 × 10^²^ copies/μL.

2.4×10^2^ copies/μL				Mean Cq	SD
BKoV	33.31	32.83	33.22	33.12	0.255
BoAstV	31.02	30.25	30.51	30.59	0.392
BToV	33.69	33.95	33.56	33.73	0.199

2.4×10^1^ copies/μL				Mean Cq	SD
BKoV	36.51	36.24	36.99	36.58	0.380
BoAstV	33.71	34.56	34.11	34.13	0.425
BToV	36.92	36.33	36.74	36.66	0.302

Cq represents cycle quantity, SD represents standard deviation, BKoV means bovine kobuvirus, BoAstV means bovine astrovirus, and BToV means bovine torovirus.

### 3.4 Specificity of the mRT-qPCR

**Figure 3:**
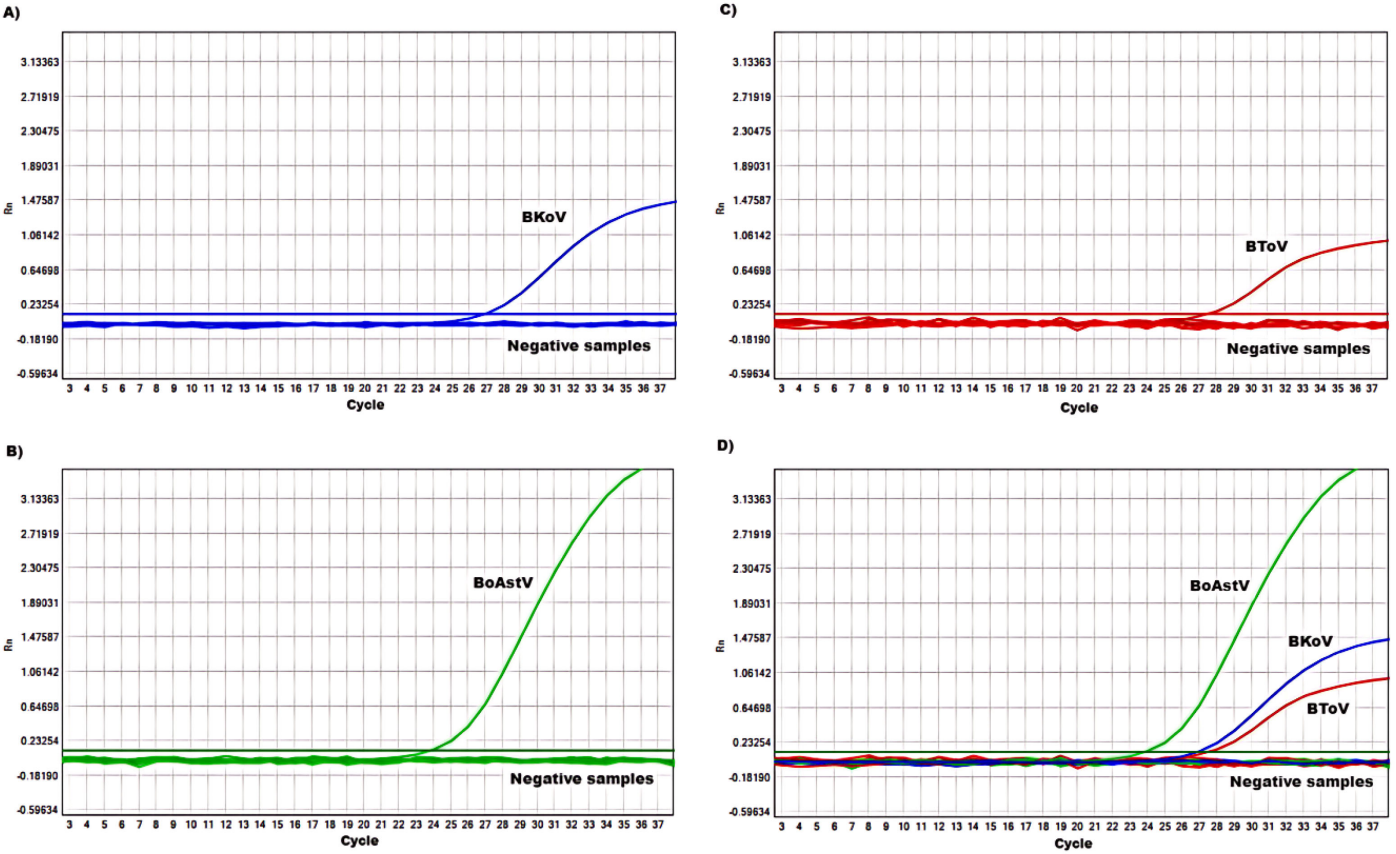
Specificity of the mRT-qPCR assay: **(A-D)** amplification curves (X-axis: Cycle, Y-axis: Rn) represent samples positive for **(A)** BKoV, **(B)** BoAstV, **(C)** BToV and **(D)** BKoV+BoAstV+BToV detected by our mRT-qPCR assay; negative samples include BVDV, BCoV, BRV, BPV, BAdV, BNoV, and negative control. BKoV means bovine kobuvirus, BoAstV means bovine astrovirus, and BToV means bovine torovirus, BVDV means bovine viral diarrhea virus, BCoV means bovine coronavirus, BRV means bovine respiratory virus, BPV means bovine parvovirus, BAdV means bovine adenovirus, BNoV means bovine norovirus.

The specificity of the mRT-qPCR assay was tested by analyzing its performance in the presence of positive samples for BKoV, BoAstV, and BToV, alongside fecal samples from calves diagnosed with diarrhea that tested positive for other viral agents, including bovine viral diarrhea virus (BVDV), bovine coronavirus (BCoV), bovine respiratory virus (BRV), bovine parvovirus (BPV), bovine adenovirus (BAdV), and bovine norovirus (BNoV). The assay successfully detected relevant fluorescent signals exclusively for the targeted viruses, with no cross-reactivity observed with the non-target viruses (**Figure 3**). These results validate the use of the primers and probes for accurate and specific detection of BKoV, BoAstV, and BToV, making the assay a reliable tool for clinical diagnostics and viral surveillance in cattle populations.

### 3.5 Reproducibility of the mRT-qPCR

The reproducibility of the mRT-qPCR assay was systematically evaluated using 10-fold serial dilutions of the standard plasmid, ensuring reliability and consistency across experimental runs. The coefficients of variation (CVs) for both intra-assay and inter-assay analyses are summarized in **Table 4**. For bovine kobuvirus (BKoV), the CVs between runs ranged from 0.08% to 0.66%; for bovine astrovirus (BoAstV), they ranged from 0.09% to 0.97%; and for bovine torovirus (BToV), the CVs extended from 0.15% to 1.29%. These low CV values indicate that the mRT-qPCR assay demonstrates excellent repeatability and high accuracy for both inter-assay and intra-assay testing, confirming the robustness and reliability of the assay in various laboratory conditions.

**Table 4:** Intra- and inter-assay variability of the mRT-qPCR assay, expressed as coefficients of variation (CV%) across multiple replicates for BoAstV, BKoV, and BToV.

Pathogen name	Template concentration (copies/μL)	Intra-assay			Inter-assay		
		Mean Cq	SD	CV (%)	Mean Cq	SD	CV (%)
BKoV	2.4×10^6^	19.953	0.035	0.18	19.637	0.015	0.08
	2.4×10^5^	23.957	0.035	0.15	24.543	0.096	0.39
	2.4×10^4^	26.950	0.026	0.10	26.717	0.110	0.41
	2.4×10^3^	30.357	0.127	0.42	31.437	0.208	0.66
BoAstV	2.4×10^6^	17.077	0.015	0.09	17.090	0.135	0.79
	2.4×10^5^	21.047	0.015	0.07	22.057	0.193	0.87
	2.4×10^4^	24.040	0.157	0.65	24.200	0.193	0.80
	2.4×10^3^	27.250	0.121	0.44	28.883	0.280	0.97
BToV	2.4×10^6^	20.453	0.071	0.35	20.553	0.221	1.07
	2.4×10^5^	24.643	0.078	0.32	25.290	0.223	0.88
	2.4×10^4^	27.273	0.040	0.15	27.573	0.097	0.35
	2.4×10^3^	30.340	0.105	0.35	32.023	0.414	1.29

Cq represents cycle quantity, SD represents standard deviation, CV represents coefficient of variation, BKoV means bovine kobuvirus, BoAstV means bovine astrovirus, and BToV means bovine torovirus.

### 3.6 Evaluation of mRT-qPCR with clinical samples

**Figure 4:**
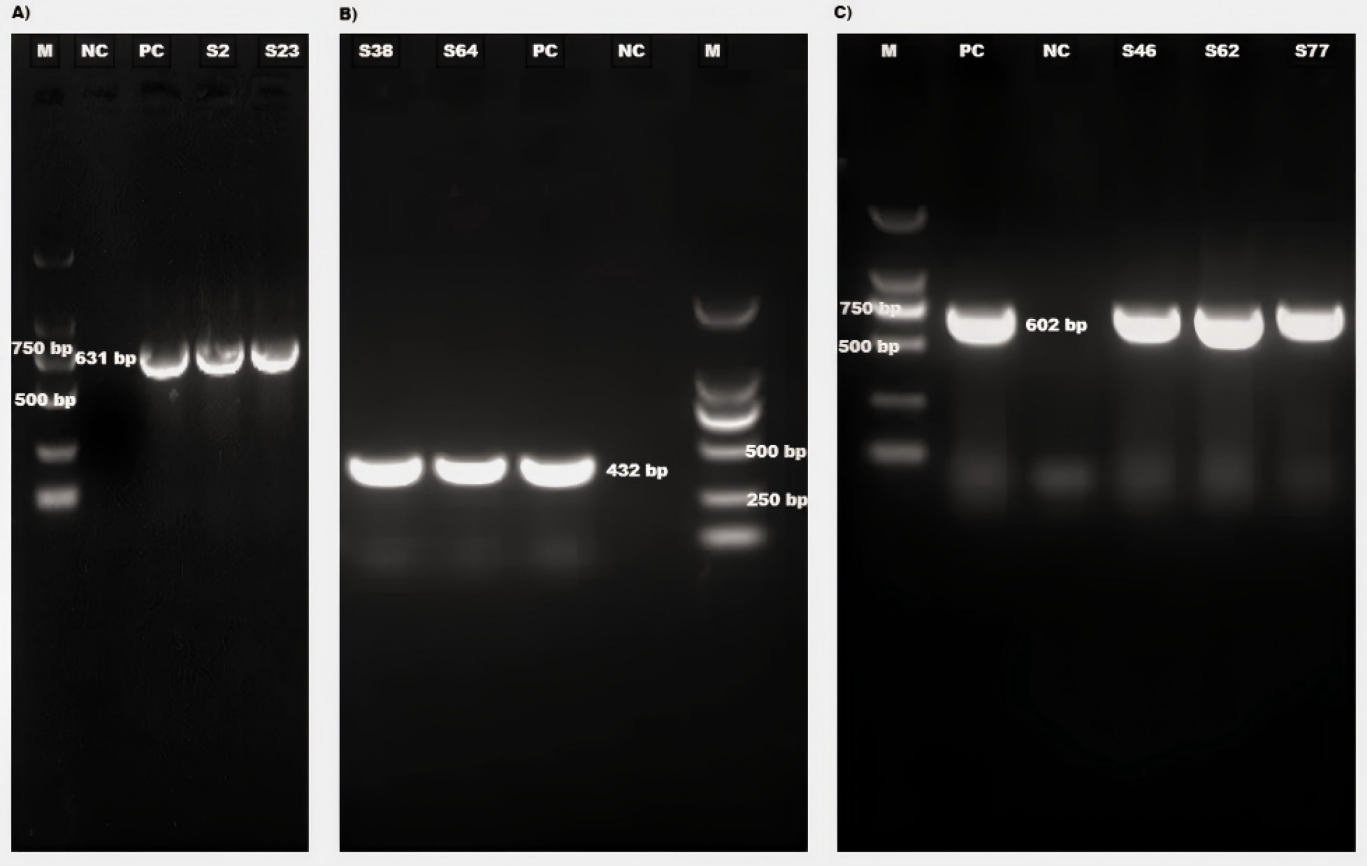
Agarose gel electrophoresis of RT-PCR products **(A)** Detection of some BKoV positive samples (product size 631 bp). **(B)** Detection of some BAostV positive samples (product size 432 bp). **(C)** Detection of BToV positive samples (product size 602 bp). M: DL2,000 DNA Marker (Takara, Beijing, China, Cat#3427A), NC, Negative Control; PC, Positive Control; S, Sample No.

To further validate the clinical applicability of the developed mRT-qPCR, 80 bovine fecal samples were tested and the results were compared with those obtained using conventional RT-PCR (**Figure 4**). Among the 80 samples, 23 were positive for BKoV using mRT-qPCR, while conventional RT-PCR detected BKoV in 22 samples. Notably, the single sample that showed an inconsistent result between the two methods had a higher Ct value (35.87), indicating a lower viral load. This suggests that the mRT-qPCR assay is more sensitive in detecting low amounts of viral RNA compared to conventional RT-PCR. For BoAstV and BToV, the results were fully consistent between the mRT-qPCR and singleplex conventional RT-PCR assays, further demonstrating the accuracy and reliability of the multiplex assay.

**Figure 5:**
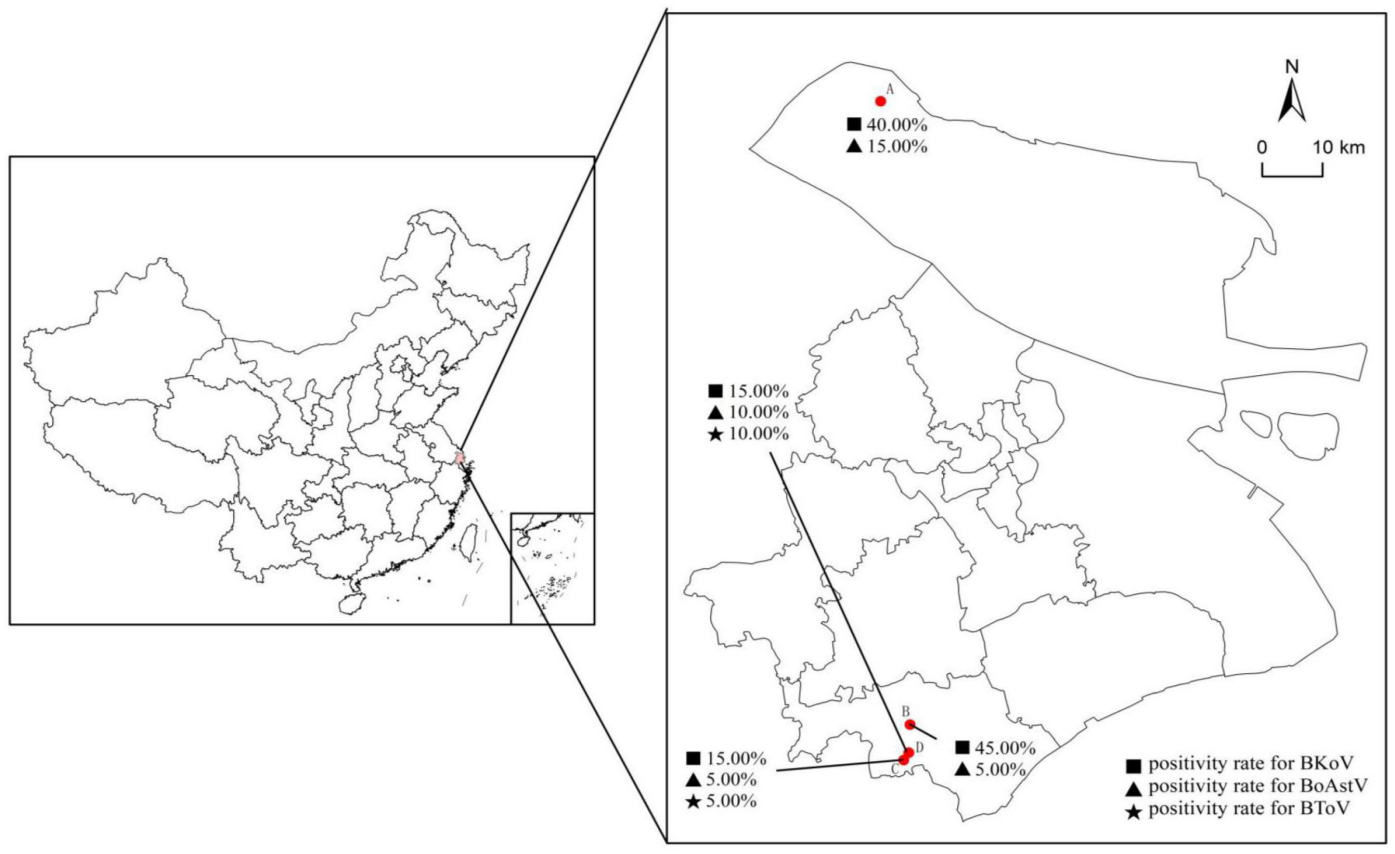
Geographical distribution of the four dairy farms (A–D) with enteric pathogens positivity rates in Shanghai. A total of 80 samples collected from four dairy farms (A–D) in Shanghai were examined for the presence of BKoV, BoAstV, and BToV. Each symbol represents a pathogen, and the percentages provided indicate the proportion of positive samples for the specified pathogens in each farm. The scale in the top right corner represents the theoretical distances on the map.

**Figure 6:**
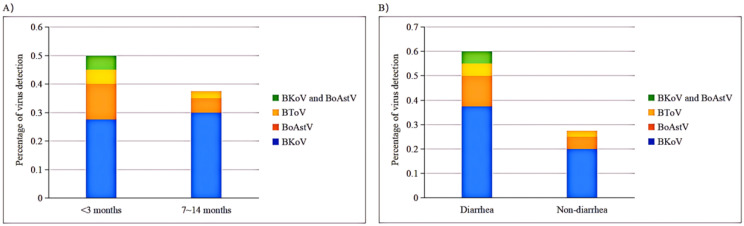
mRT-qPCR based detection rates of each pathogen based on various age groups or diarrheal status. **(A)** Virus detection in various age groups. **(B)**Virus detection in diarrheal status.

Nucleic acid detection for BKoV, BoAstV, and BToV was conducted by mRT-qPCR on all 80 samples from four dairy farms (A-D) in Shanghai (**Figure 5**). The detection rates of the viral pathogens in the clinical samples are summarized in **Table 5**. The mRT-qPCR assay identified 23 samples (28.75%) as positive for BKoV, 7 samples (8.75%) as positive for BoAstV, and 3 samples (3.75%) as positive for BToV. The association between viral pathogens and calf age was investigated (**Figure 6A**). When analyzing the results based on calf age, calves younger than 3 months showed the highest rate of infections. BKoV, BoAstV, and BToV were detected in 11 (27.50%), 5 (12.50%), and 2 (5.00%) samples, respectively, from calves younger than 3 months. For calves aged between 7 to 14 months, BKoV, BoAstV, and BToV were detected in 12 (30.00%), 2 (5.00%), and 1 (2.50%) samples, respectively. There were no statistically significant differences in viral detection based on the age of the calves, suggesting that these pathogens may affect calves across different age groups without a strong age-related prevalence pattern. The BoAstV detection rate in dairy feces was higher in calves aged 3 months compared with calves aged between 7 and 14 months. The differences in the detection rates of viral pathogens according to clinical symptoms (diarrhea or non-diarrhea) were also investigated (**Figure 6B**). Among the 40 fecal samples collected from diarrheic calves, BKoV, BoAstV, and BToV were detected in 15 (37.50%), 5 (12.50%), and 2 (5.00%) samples, respectively. In contrast, among the 40 samples from healthy calves, the detection rates were 8 (20.00%) for BKoV, 2 (5.00%) for BoAstV, and 1 (2.50%) for BToV. These results suggest that while there is a higher detection rate of these viruses in diarrheic calves, there was also no statistically significant association between the presence of these viral pathogens and the occurrence of diarrhea. The diarrheic calves showed the highest rate of infections. In the present study, five BoAstV-positive cases were associated with clinical diarrhea in cattle where calves aged < 3 month accounted for 80% of cases (**Table 5**). Moreover, BToV can be detected occasionally in healthy calves. As shown in **Figure 6**, co-infections with BKoV + BoAstV were detected in two diarrheic calves aged < 3 months. These findings indicate that the mRT-qPCR assay is a highly sensitive and specific method for detecting BKoV, BoAstV, and BToV in clinical samples, providing a useful tool for the rapid diagnosis and monitoring of viral pathogens associated with calf diarrhea.

**Table 5:** Summary of detection results for bovine fecal samples using the developed mRT-qPCR assay, indicating the presence of BKoV, BoAstV, and BToV in clinical samples from diarrheic and non-diarrheic calves.

Pathogens	Age group (month)	Overall % positive	% positives among diarrheic calves	% positives among healthy calves	*P* value
BKoV	<3	27.50 (11/40)	35.00 (7/20)	20.00 (4/20)	0.2881
	7~14	30.00 (12/40)	40.00 (8/20)	20.00 (4/20)	0.1675
	Total	28.75 (23/80)	37.50 (15/40)	20.00 (8/40)	0.0837
BoAstV	<3	12.50 (5/40)	20.00 (4/20)	5.00 (1/20)	0.3416
	7~14	5.00 (2/40)	5.00 (1/20)	5.00 (1/20)	1.000
	Total	8.75 (7/80)	12.50 (5/40)	5.00 (2/40)	0.4315
BToV	<3	5.00 (2/40)	5.00 (1/20)	5.00 (1/20)	1.000
	7~14	2.50 (1/40)	5.00 (1/20)	0.00 (0/20)	1.000
	Total	3.75 (3/80)	5.00 (2/40)	2.50 (1/40)	1.000

## 4 Discussion

Calf diarrhea represents a significant challenge within the cattle industry, leading to substantial economic losses and affecting the overall health and productivity of livestock (Kim et al., 2021; Jessop et al., 2024). As a multifactorial disease, calf diarrhea is often precipitated by a combination of viral and bacterial pathogens (Robi et al., 2024; Wang et al., 2024). Evidence suggests that either single or concurrent infections by viral agents are critical contributors to the development of this condition. Numerous pathogenic viruses have been well-documented in cattle, each capable of inducing a spectrum of clinical manifestations ranging from acute to persistent or latent infections (Gomez and Weese, 2017). Although instances of acute mortality following viral infections are relatively uncommon, these viruses typically exhibit characteristics such as prolonged infection durations, rapid transmission rates, and extensive dissemination, which can precipitate large-scale outbreaks and exacerbate economic consequences. The clinical similarities and overlapping transmission routes among these pathogens often result in mixed infections, complicating clinical diagnosis and exacerbating the severity of diarrhea, which in turn heightens mortality risk. Consequently, early and accurate diagnosis of calf diarrhea is of paramount economic and clinical importance.

Currently, quantitative PCR (qPCR) is widely regarded as one of the most effective methodologies for the molecular detection of pathogens, owing to its reliability, sensitivity, and cost-effectiveness (He et al., 2023; Liu et al., 2023). However, conventional qPCR is predominantly suited for the detection of single pathogens, which can be time-consuming and inefficient in cases where multiple pathogens are involved. This limitation underscores the urgent need for multiplex detection methods capable of identifying concurrent infections rapidly and economically. Multiplex real-time polymerase chain reaction (mRT-qPCR) has emerged as a promising alternative, allowing for enhanced detection capabilities while reducing the time and labor costs associated with traditional RT-PCR techniques (Amirudeen and Paret, 2024; Belem et al., 2024; Shin et al., 2024). Given that bovine diarrheal disease syndromes are frequently driven by co-infections involving multiple pathogens, the development of a rapid and convenient multiplex assay is critical for timely and effective diagnostics.

In this study, we successfully developed a novel mRT-qPCR assay using molecular beacon-based gene (MBG) probes to simultaneously detect bovine kobuvirus (BKoV), bovine astrovirus (BoAstV), and bovine torovirus (BToV) in a single reaction. The specificity of the probes was confirmed through BLAST analysis, which demonstrated their exclusivity for the targeted viral species. To optimize the performance of our mRT-qPCR assay, we meticulously evaluated several parameters, including analytical sensitivity, specificity, and reproducibility. The limit of detection (LOD) achieved for our assay was notably low, at 24 copies/μL for all three viral targets, demonstrating a substantial increase in sensitivity—by 10 to 10,000 times—compared to other conventional RT-PCR methods (Zhou et al., 2017; Pan et al., 2020; Yang et al., 2020). Furthermore, through the analysis of 80 clinical samples, our assay identified positive cases that had previously been overlooked as negative by conventional RT-PCR. Notably, the LOD for BKoV and BoAstV in our mRT-qPCR assay surpassed that of singleplex RT-qPCR assays (Lüthi et al., 2018; Liu et al., 2022), while the LOD for BToV remained consistent across both methodologies (Zhao et al., 2021).

The amplification efficiency observed from the standard plasmid was within the recommended range of 90–110%, and the linear regression coefficient (R²) for all standard curves was greater than 0.996, indicating a robust linear relationship and reliability of the assay. Importantly, our assay exhibited no cross-reactivity with other viral pathogens typically associated with diarrhea, such as bovine viral diarrhea virus (BVDV), bovine coronavirus (BCoV), bovine respiratory virus (BRV), bovine parvovirus (BPV), bovine adenovirus (BAdV), or bovine norovirus (BNoV). Additionally, the coefficients of variation (CVs) for both intra-assay and inter-assay replicates were consistently below 1.5%, attesting to the repeatability and stability of our method.

Subsequently, the established mRT-qPCR assay was utilized to evaluate 80 fecal samples collected from four dairy farms in Shanghai, China, revealing positive detection rates of 28.75% for BKoV, 8.75% for BoAstV, and 3.75% for BToV. The co-occurrence of BoAstV with other enteric viruses has been reported since its discovery (Zhu et al., 2022), while the positivity rate of co-infection with BKV was the highest, at up to 66.67% (Oem and An, 2014; Zhu et al., 2021). The present study detected 7 cases of BoAstV positivity, with 2 cases showing co-infection with BKoV, consistent with previous studies (Oem and An, 2014; Zhu et al., 2021). This study marks the first detection of these viruses within the Shanghai region, indicating their prevalence and potential impact on local cattle populations. The higher frequency of positive samples identified in diarrheic calves compared to non-diarrheic counterparts aligns with previous findings within the literature (Kirisawa et al., 2007; Candido et al., 2017; Zhu et al., 2021). Furthermore, the detection of a greater number of BoAstV-positive samples in calves under three months of age as compared to those aged between 7 and 14 months highlights the importance of age as a factor in susceptibility to viral infections (Oem and An, 2014).

## 5 Conclusions

In summary, the mRT-qPCR assay developed in this study is a highly specific, sensitive, and reproducible tool for the simultaneous detection of BKoV, BoAstV, and BToV. Its application provides a robust, rapid, and simple method for the differential diagnosis of calf diarrhea caused by these viral pathogens. This multiplex assay is not only a valuable diagnostic tool but also offers significant benefits in terms of reducing labor, time, and cost in clinical and epidemiological settings. The implementation of this method in clinical settings is anticipated to not only augment detection capabilities but also alleviate the workload on veterinary practitioners and epidemiologists, ultimately benefiting livestock health and productivity in the cattle industry. Our results will contribute to better understanding of these challenging gastrointestinal viruses and may help to spur studies designed to prevent and control calf diarrhea in China.

## Data Availability

The original contributions presented in the study are included in the article/supplementary material. Further inquiries can be directed to the corresponding author/s.
